# Influence of Ethnic Group-Membership and Gaze Direction on the Perception of Emotions. A Cross-Cultural Study between Germany and China

**DOI:** 10.1371/journal.pone.0066335

**Published:** 2013-06-07

**Authors:** Katharina Krämer, Gary Bente, Siyang Luo, Ulrich J. Pfeiffer, Shihui Han, Kai Vogeley

**Affiliations:** 1 Neuroimaging Group, Department of Psychiatry, University Hospital Cologne, Germany; 2 Department of Psychology, University of Cologne, Cologne, Germany; 3 Culture and Social Cognitive Neuroscience Lab, Department of Psychology, Beijing University, Beijing, China; 4 Institute for Neuroscience and Medicine – Cognitive Neuroscience (INM3), Research Center Juelich, Juelich, Germany; Ecole Normale Supérieure, France

## Abstract

Emotional facial expressions provide important nonverbal cues in human interactions. The perception of emotions is not only influenced by a person’s ethnic background but also depends on whether a person is engaged with the emotion-encoder. Although these factors are known to affect emotion perception, their impact has only been studied in isolation before. The aim of the present study was to investigate their combined influence. Thus, in order to study the influence of engagement on emotion perception between persons from different ethnicities, we compared participants from China and Germany. Asian-looking and European-looking virtual agents expressed anger and happiness while gazing at the participant or at another person. Participants had to assess the perceived valence of the emotional expressions. Results indicate that indeed two factors that are known to have a considerable influence on emotion perception interacted in their combined influence: We found that the perceived intensity of an emotion expressed by ethnic in-group members was in most cases independent of gaze direction, whereas gaze direction had an influence on the emotion perception of ethnic out-group members. Additionally, participants from the ethnic out-group tended to perceive emotions as more pronounced than participants from the ethnic in-group when they were directly gazed at. These findings suggest that gaze direction has a differential influence on ethnic in-group and ethnic out-group dynamics during emotion perception.

## Introduction

The expression of emotions has a vital communicative and social function [1], [2] and is an important aspect of nonverbal communication [3], [4]. In social interactions, the expression of emotions is substantially influenced by the relationship between the interaction partners [5] and can convey affiliation and dominance [6]. In addition, the perception of a person and their emotional expressions underlies the influence of social engagement. Recent proposals of a ‘second-person’ approach to social cognition highlight the importance of interacting with someone in order to understand and predict her/his behaviour [7], [8]. These proposals suggest a difference in the perception of others depending on whether we are actively engaged with another person or whether we are passive observers of an interaction [9]. Schilbach and colleagues [10] investigated this difference by varying the gaze direction of a virtual agent with whom participants interacted. Indeed, results demonstrated that participants felt more engaged with the virtual agent when they were directly gazed at as compared to when they observed a virtual agent gazing at another person. Another study demonstrated that gaze direction facilitates the recognition of emotions [11]. In particular, this study showed that approach-orientated emotions (anger and happiness) were better recognised with direct gaze than with averted gaze. Taken together, these findings suggest that gaze direction does not only provide an important social signal, but offers contextual information that is critical for the interpretation of behavioural intentions conveyed by emotional expressions.

Besides that, the perception of emotional expressions is also of great interest from a cultural perspective. Previous research suggests that there are cultural universalities in the expression and recognition of emotions which enable people to recognize emotional expressions shown by members of different ethnic groups [12]–[14]. However, it has also been shown that the perception of emotional expressions is influenced by the ethnic background of an interaction partner - i.e. the perceiver’s feeling of belonging to the same (“ethnic in-group”) or a different ethnic group (“ethnic out-group”). In their study, Hess and colleagues [15] investigated the influence of facial emotion displays (happiness and anger) and ethnicity on dominance and affiliation judgements. They expected the ethnic background of the interaction partners to play an important role in the attribution of behavioural intentions based on cultural stereotypes. In particular, they hypothesized that the perceived likelihood for an expression to be shown by members of a specific ethnic group would have an influence on observers’ ratings of dominance and affiliation. Their results support this hypothesis: North Americans were rated as more likely to show anger than Asians. In addition, they were rated as more dominant compared to Asians, thereby indicating that the likelihood of the anger display predicted dominance ratings. Furthermore, Asians were rated as more likely to show happiness than North Americans. Accordingly, happiness displays by Asians were rated as more affiliative. In another study, Brown and colleagues [16] investigated emotional responses to pictures of ethnic in-group and out-group members which varied in pleasantness. They found that people experienced greater pleasure and displeasure and thus responded more extremely when viewing pictures of ethnic in-group as compared to ethnic out-group members. They concluded that in-group dynamics exert a greater influence on affective reactions than out-group dynamics.

Apart from gaze direction and ethnicity, the type of stimulus material and its visualization plays an essential role in the study of emotional expressions. Over the last years, it has been shown that the investigation of emotions heavily depends on the dynamic characteristics of the stimulus material used. Several studies indicated that dynamic emotional expressions do not only offer an advantage in the intensity evaluation and recognition of emotions when compared to static emotional expressions, but also increase participants’ reactions to emotional expressions. It has been shown that intensity ratings are higher and recognition rates are better for animations of emotional expressions than for photographs of emotional expressions [17], [18]. In addition, electromyographical recordings of facial muscular reactions suggested stronger reactions to dynamic as compared to static emotional expressions [19], [20]. Past research has suggested that anthropomorphic virtual agents are particularly suited to implement and visualize such dynamic emotional expressions, because their nonverbal behaviour and their outward appearance can be controlled and varied systematically [21]–[23]. Several studies have provided evidence that virtual agents’ nonverbal behaviour is perceived in much the same way as the nonverbal behaviour of real humans [24], [25]. In addition, studies in so-called ‘shared virtual environments’ have repeatedly shown that interactions with virtual agents follow the same social norms as social interactions with real persons [26]–[28]. As a consequence, the last years have seen a rise in the use of virtual agents to study human behaviour in interactions [29]–[32]. Most importantly for the present study, it has been demonstrated that virtual and natural emotional expressions are recognised to a comparable degree [21]. Intriguingly, not only the passive detection of emotions is comparable to real-life encounters, but also the effect of emotional contagion occurs in interactions with virtual agents: Using electromyographical recordings of facial muscular reactions, it could be demonstrated that participants show facial mimicry of virtual agents’ emotional expressions similar to human-human interactions [33]–[35]. In sum, it has therefore been concluded that virtual agents “can be used as well-controlled, realistic and dynamic stimuli in emotion research” ([21], p. e3628).

The present study is particularly motivated by a review of Wieser and Brosch [36], who emphasized that emotions are mostly perceived within a situational context and should therefore be studied within such a context. This situational context depends on different factors including features of the encoder of an emotion (e.g. gaze direction and expression dynamics), personal aspects concerning the perceiver of an emotion (e.g. ethnicity), and the common physical environment. As argued above, the individual influences of different factors on emotion perception could already be shown repeatedly over the last years. However, according to Wieser and Brosch, these factors are interdependent, so that contemporary research on emotion perception should also address the interactions between these factors [36].

**Figure 1:**
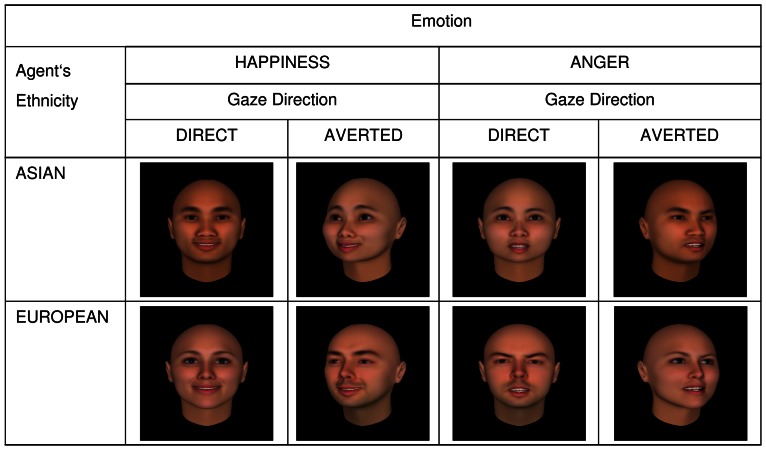
Experimental paradigm with factors gaze direction, agent’s ethnicity, and emotion. *****Note:* Between-subject variable  =  participant’s ethnicity.****

On this background, we chose to investigate the interaction of ethnicity and gaze direction in the present study. Both factors are nonverbal and can systematically be varied using virtual agents, which offers the opportunity of combining them in one stimulus set. In order to include participants from two distinct ethnic groups, we decided to compare subjects from the Eastern (China) and the Western (Germany) culture, as these cultures are known to differ in cultural constructs such as individualism and collectivism [37], [38]. Based on a previous study of our group [10], we designed a four-factorial experimental paradigm. Firstly, participants’ engagement with a virtual agent (hereinafter referred to as ‘agent’) was modulated. Agents either gazed directly at the participants or at a third invisible person who was situated at an angle of approximately °20 besides the participants. Secondly, agents displayed two distinct dynamic emotional expressions, happiness and anger, which had to be assessed by the participants with respect to their valence. Thirdly, agents either appeared Asian or European to manipulate participants’ sense of belonging either to the ethnic in- or out-group. Finally, participant’s ethnicity, Chinese or German, was the between subject factor. The four-factorial design including the factors (i) *gaze direction* (DIRECT versus AVERTED), (ii) *emotion* (ANGER versus HAPPINESS), (iii) *agent’s ethnicity* (ASIAN versus EUROPEAN), and (iv) *participant’s ethnicity* (CHINESE versus GERMAN) is depicted in Figure 1.

Based on the literature discussed above, we hypothesized that emotion appraisal is influenced both by the participants’ and the agents’ ethnic group-membership, and, more precisely, that emotions are rated more extremely when the participant and the agent are members of the same ethnic group [16]. Additionally, we hypothesized that participants rate the valence of emotions more extremely when displayed in combination with direct gaze as compared to averted gaze [11]. Finally, we hypothesized an interaction between ethnicity and gaze direction, such that the effect of gaze direction on emotion perception will be more pronounced when the participant and the agent belong to the same ethnic group.

## Materials and Methods

### Ethics Statement

The study was approved by the local ethics committee of the Medical Faculty of the University of Cologne, Germany. Written informed consent was obtained from all participants.

### Participants

A group of 40 students at Beijing University in China (20 female, 20 male), and a group of 40 students at the University of Cologne in Germany (20 female, 20 male) volunteered to participate in the study. All Chinese participants (M = 22.38 years; SD  = 2.168 years) were born and raised in China, all German participants (M = 23.93 years; SD  = 4.736 years) were born and raised in Germany. There was no significant difference in age between Chinese and German participants (*t* (1, 78)  = 1.882, *p*>.05). All participants were naive concerning the purpose of the study.

### Stimulus Material

The software package FaceGen (© Singular Inversions Inc., Toronto, Canada, 2012) was used to create the stimulus material. This software allows generating three-dimensional (3D) agents from photographs of real persons. We used this function to create 20 faces of Asian-looking agents (10 female, 10 male) and 20 faces of European-looking agents (10 female, 10 male) based on photographs of Chinese and German persons (see Figure 1). All participants who provided their picture in order to generate 3D agents gave written informed consent, as outlined in the PLOS consent form, to publication of the agent based on their picture. Further, three-second long animations of emotional expressions were created using the virtual reality software Vizard (©WorldViz Inc, Santa Barbara, USA, 2012). Each agent was presented four times: expressing anger and happiness while gazing directly at the participants and while averting its gaze towards another person. All agents, independently of their ethnic background, showed the identical angry and happy expression. Accordingly, participants saw a total of 160 animations of emotional facial expressions (in each case, 20 European-looking and 20 Asian-looking agents showed anger with direct gaze and averted gaze, and happiness with direct gaze and averted gaze).

A pilot study was conducted for external validation of the stimulus material. 20 students from Germany (female  =  12; all born and raised in Germany) and 12 students from China (female  =  5; all born and raised in China) participated in the pilot study. Firstly, participants were asked to assess the ethnicity and gender of each agent. Results indicated that all agents were categorised correctly concerning their ethnicity and gender by participants from both ethnic groups. Secondly, the emotional facial expressions were assessed. For this purpose, participants had to categorise three-second long video animations of Asian and European agents showing anger, fear, sadness, happiness, and surprise. The results showed that anger (78% by German participants; 83% by Chinese participants) and happiness (94% by German participants; 92% by Chinese participants) were correctly categorised by the participants. Thus, for the present study, we assume that happiness and anger can be recognized correctly by participants from both ethnic groups.

### Procedure

Participants were seated in front of a computer screen where they watched the video sequences. They were instructed that they would see agents expressing different emotions while either gazing directly at them or averting their gaze towards another person. In the former case, the agent would look directly at the participants while expressing the emotions. In the latter case, the agent would be rotated to the left or the right side at an angle of 20° (see Figure 1) and direct an emotional expression towards a putative second person who was effectively invisible to the participants [10]. Participants were instructed to imagine this second person standing on the left or the right side behind them. After each video sequence, participants had to assess the valence of the expressed emotion of the agent on a four-point rating scale (1 =  negative; 2 =  rather negative; 3 =  rather positive; 4 =  positive). Following the experiment, participants saw a picture of each agent presented in the study. For each agent they had to categorise its ethnicity. Results indicate that all agents were categorised correctly concerning their ethnicity. In addition, we measured the cultural constructs of individualism and collectivism using Singelis’ Self-Construal Scale [39]. Results indicate that Chinese participants (M = 5.17; SD  = .48) scored higher than German participants (M = 4.42; SD  = .64) on the subscale measuring collectivism, *t* (1, 78)  = 5.696, *p* = .000. In contrast, German participants (M = 5.1; SD = .57) scored higher than Chinese participants (M = 4.68; SD = .54) on the subscale measuring individualism, *t* (1, 78)  = –3.159, *p* = .002.

### Data Analyses

All data were analyzed using IBM SPSS Statistics 20 (SPSS Inc, Chicago, IL, 2011). A mixed ANOVA with the between-group factor *participant’s ethnicity* and the three repeated-measures variables *agent’s ethnicity*, *emotion*, and *gaze direction* was conducted. Planned simple comparisons (Bonferroni-corrected) were computed to break down interaction effects. To analyze the effects of gender, a mixed ANOVA with the between-group factors *participant’s gender* and *participant’s ethnicity* and the four repeated-measures variables *agents’ gender*, *agents’ ethnicity*, *emotion*, and *gaze direction* was conducted. For main effects, interaction effects, and planned simple comparisons, Pearson’s correlation coefficient (*r*) is reported as a measure of effect size [40]. The following conventions for interpreting *r* are suggested: Small effect: *r* = .10; medium effect: *r* = .30; large effect: *r* = .50 [40]. Bivariate correlation analyses with Pearson’s product-moment correlation coefficient (*r*) [40] were conducted in order to test whether there was a statistical relationship between participants’ ratings on Singelis’ Self-Construal Scale [39] and the behavioural effects. For this purpose, a *SCS index* for each participant was calculated: Participants’ mean agreement for the individualistic items was subtracted from participants’ mean agreement for the collectivistic items. Accordingly, participants with a positive SCS index showed more agreement for collectivistic items, while participants with a negative SCS index showed more agreement for individualistic items.

## Results

Chinese and German participants did not differ in their emotion appraisal in general, *F* (1,76) = 1.374, *p* = .245. Additionally, there was no significant difference in the assessment of the valence of the emotions between female and male participants (*F*(1, 76)  = .066, *p* = .797). Concerning the *agent’s gender* a significant difference was found, *F*(1, 76)  = 39.818, *p* = .000, *r* = .344, indicating that female agents (M = 2.599; SD  = .034) are assessed generally more positive than male agents (M = 2.522; SD  = .03). However, there was no significant interaction effect of *agent’s gender* with any of the other factors, which justified the exclusion of the factor *gender* from further analyses.

### Effects of ethnicity

A significant main effect of *agent’s ethnicity* (*F*(1, 78)  = 55.249, *p* = .000, *r* = .644) suggested that Asian agents (*M* = 2.601; *SD*  = .13) were rated as more positive than European agents (*M* = 2.52; *SD* = .126). Furthermore, a significant interaction effect between *participant’s ethnicity* and *agent’s ethnicity* was found (*F*(1, 78)  = 14.842, *p* = .000, *r* = .400). Simple comparisons revealed that Chinese and German participants did not differ in their appraisal of European agents, *F* (1,78)  = .054, *p*>.05 (Chinese participants: *M* = 2.516, *SD* = .094; German participants: *M* = 2.523, *SD*  = .116). However, both groups differed in their appraisal of Asian agents (*F*(1, 78)  = 5.764, *p* = .019, *r* = .262): Chinese participants (*M* = 2.639; *SD*  = .108) assessed Asian agents more positively than German participants (*M* = 2.563, *SD* = 0.112). To assess whether the cultural constructs of individualism and collectivism were related to participants’ ratings of the video sequences, correlation analyses of *participants’ SCS indices* and the assessment of *Asian agents* and *European agents* were conducted. There was a significant relationship between *participants’ SCS indices* and the assessment of *Asian agents*, *r* = .21, *p* = .033. The more positive the SCS index of a participant was (i.e. the more agreement she/he showed for collectivistic items), the more positive she/he assessed Asian agents. However, *participants’ SCS indices* were not significantly related to the assessment of *European agents*, *r* = –.04, *p* = .359.

### Effects of gaze direction

A significant interaction effect of *emotion* and *gaze direction* was found, *F*(1, 78)  = 17.518, *p* = .000, *r* = .433. Simple comparisons showed that ANGERxDIRECT (*M* = 1.621; *SD* = .116) was rated more negatively (*F*(1, 78)  = 9.783, *p* = .002, *r* = .334) than ANGERxAVERTED (*M* = 1.683; *SD* = .101). Further, HAPPINESSxDIRECT (*M* = 3.518; *SD*  = .108) was rated more positively (*F*(1, 78)  = 17.934, *p* = .000, *r* = .432) than HAPPINESSxAVERTED (*M* = 3.42; *SD* = .118).

### Interaction effects of gaze direction * ethnicity

**Figure 2:**
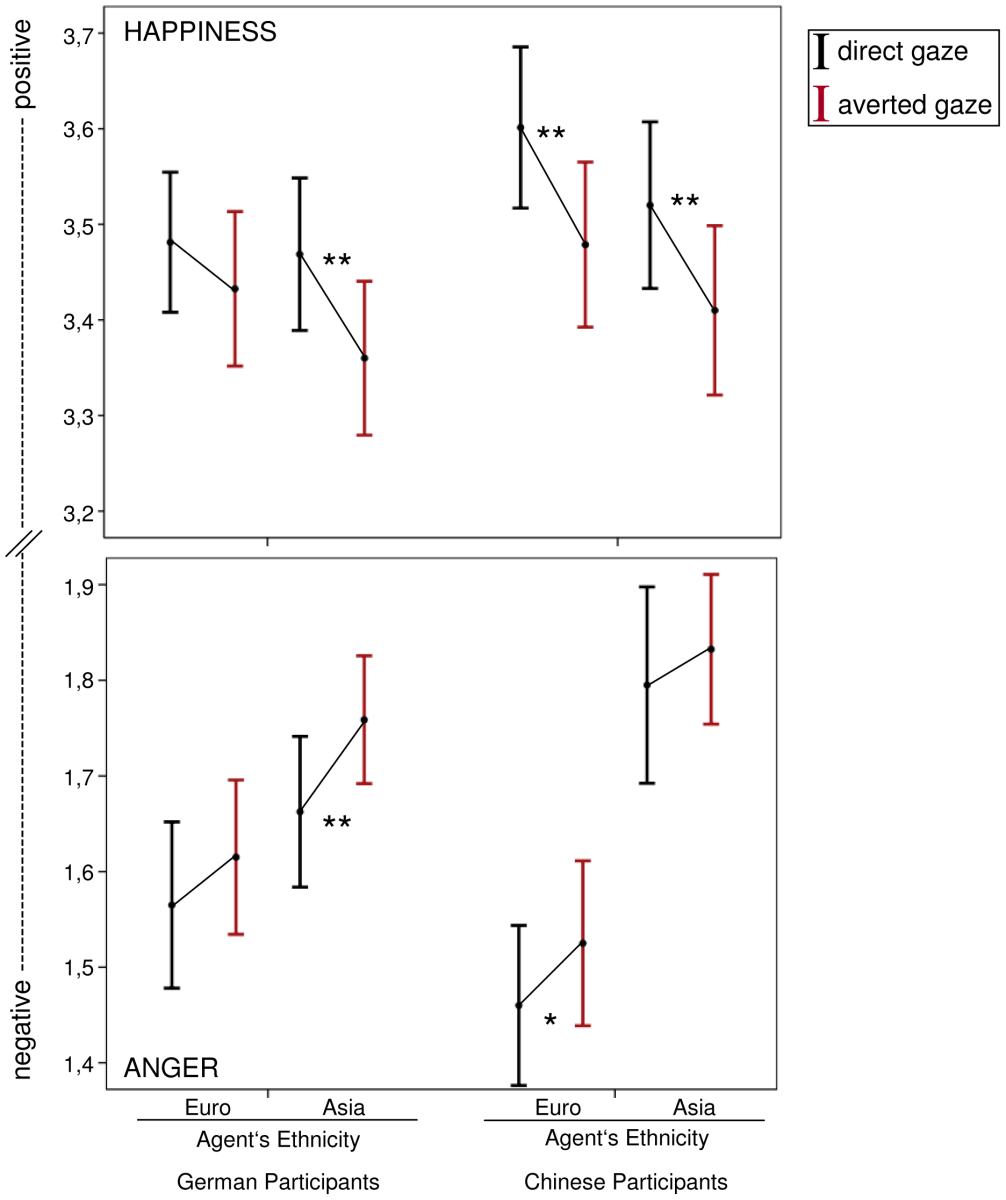
Interaction effect of gaze direction, participant’s ethnicity, agent’s ethnicity, and emotion. Top panel shows results for happiness, bottom panel shows results for anger. *Note:* Error bars indicate the 95% confidence interval. * *p*<.05; ** *p*<.01.

To analyze the influence of ethnicity on the valence appraisal of emotions expressed with direct gaze as compared to averted gaze, the four-way interaction between *gaze direction*, *participant’s ethnicity*, *agent’s ethnicity*, and *emotion* was considered. A significant interaction effect occurred, *F*(1, 78)  = 4.923, *p* = .029, *r* = .244 (see Figure 2). Simple comparisons presented in Table 1 show that Germans assess ASIANxANGERxDIRECT as more negative than ASIANxANGERxAVERTED (*F*(1, 78)  = 1.717, *p* = .007, *r* = .147). Additionally, Germans assess ASIANxHAPPINESSxDIRECT as more positive than ASIANxHAPPINESSxAVERTED (*F*(1, 78)  = 8.610, *p* = .004, *r* = .315). However, for Germans there was no difference in the appraisal of EUROPEANxANGERxDIRECT and EUROPEANxANGERxAVERTED (*F*(1, 78)  = 2.937, *p*>.05). Germans also did not show any difference in the appraisal of EUROPEANxHAPPINESSxDIRECT and EUROPEANxHAPPINESSxAVERTED (*F*(1, 78)  = 1.925, *p*>.05). Concerning Chinese participants, results show that they rated EUROPEANxANGERxDIRECT more negatively than EUROPEANxANGERxAVERTED (*F*(1, 78)  = 4.963, *p* = .029, *r* = .245). In addition, Chinese rated EUROPEANxHAPPINESSxDIRECT as more positive than EUROPEANxHAPPINESSxAVERTED (*F*(1, 78)  = 12.154, *p* = .001, *r* = .367). In contrast, Chinese showed no difference in the valence appraisal between ASIANxANGERxDIRECT and ASIANxANGERxAVERTED (*F*(1, 78)  = 1.171, *p*>.05). However, Chinese assessed ASIANxHAPPINESSxDIRECT more positively than ASIANxHAPPINESSxAVERTED (*F*(1, 78)  = 8.809, *p* = .004, *r* = .319). In order to further investigate whether *participants’ SCS indices* were related to the behavioural effects described above, correlation analyses were conducted. There was a significant relationship between *participants’ SCS indices* and the assessment of ASIANxHAPPINESSxDIRECT, *r* = .147, *p* = .03: The more positive the SCS index of a participant was (i.e. the more agreement she/he showed for collectivistic items), the more positive she/he assessed ASIANxHAPPINESSxDIRECT. However, no other correlations of *participants’ SCS indices* with the behavioural effects were significant.

**Table 1:** Simple comparisons, means and standard deviations of the interaction effect of *gaze direction, participant’s ethnicity*, *agent’s ethnicity* and *emotion.*

	German participants										Chinese participants								
	European agents					Asian agents					European agents					Asian agents			
	Anger		Happiness			Anger		Happiness			Anger		Happiness			Anger		Happiness	
	Direct	Averted	Direct	Averted		Direct	Averted	Direct	Averted		Direct	Averted	Direct	Averted		Direct	Averted	Direct	Averted
df	1		1			1		1			1		1			1		1	
df (err.)	78		78			78		78			78		78			78		78	
*F*	2.937		1.925			1.717		8.610			4.963		12.154			1.171		8.809	
*p*	>.05		>.05			**.007**		**.004**			**.029**		**.001**			>.05		**.004**	
*r*						.147		.315			.245		.367					.319	
M	1.565	1.615	3.481		3.433	1.662	1.759	3.469		3.36	1.46	1.525	3.601		3.479	1.795	1.833	3.52	3.41
SD	.272	.252	.229		.253	.258	.264	.236		.271	.261	.270	.263		.270	.321	.245	.272	.277

**Figure 3:**
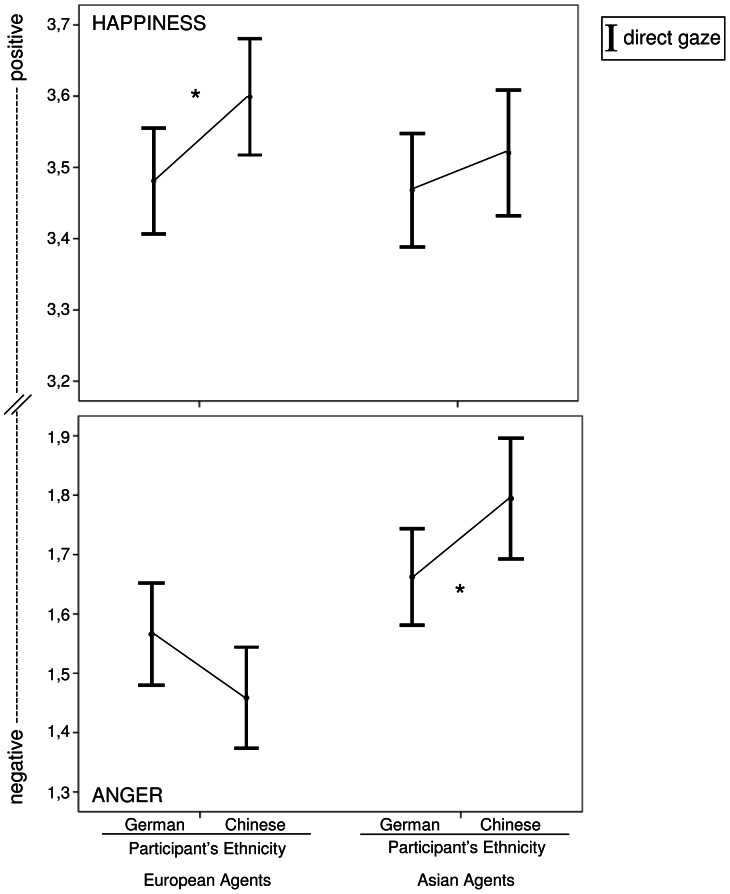
Interaction effect of participant’s ethnicity, agent’s ethnicity, gaze direction, and emotion. Results refer to agents that show direct gaze (DIRECT). Top panel shows results for happiness, bottom panel shows results for anger. *Note:* Error bars indicate the 95% confidence interval. * *p*<.05.

In addition, the significant four-way interaction between *participant’s ethnicity*, *agent’s ethnicity*, *gaze direction*, and *emotion* (*F*(1, 78)  = 4.923, *p* = .029, *r* = .244) was further examined in order to investigate the influence of the agent’s ethnicity on the emotion appraisal of Germans and Chinese. Simple comparisons did not reveal any differences in the valence appraisal between Germans and Chinese when the agents displayed an emotion while averting their gaze towards another person (*F*(1, 78)  = 2.099, *p*>.05). Therefore, the results presented below only refer to agents gazing directly at the participants (see Figure 3). There was no significant difference between Chinese and Germans in the appraisal of EUROPEANxANGERxDIRECT (*F*(1, 78)  = 3.095, *p* = .082). However, a tendency could be observed that Chinese assessed anger expressed by European agents more negatively than Germans. A significant difference between Chinese and Germans in the appraisal of EUROPEANxHAPPINESSxDIRECT was found (*F*(1, 78)  = 4.723, *p* = .033, *r* = .239): Chinese assessed happiness expressed by European agents more positively than Germans. Furthermore, there was a significant difference between Chinese and Germans in the appraisal of ASIANxANGERxDIRECT (*F*(1, 78)  = 4.288, *p* = .042, *r* = .228). Germans rated anger expressed by Asian agents more negatively than Chinese. Finally, we found no difference between Chinese and Germans in the appraisal of ASIANxHAPPINESSxDIRECT (*F*(1, 78)  = .771, *p*>.05).

## Discussion

In the present study, we investigated the interaction between ethnic group-membership and gaze direction on the perception of emotional expressions. Our results show that two factors which are known to influence emotion perception interact in their combined influence. Although previous research suggests that gaze direction influences emotion perception such that emotions are perceived as more extreme when expressed with direct gaze, our findings indicate that ethnicity influences this effect. We found that, except for Chinese assessing ASIANxHAPPINESS, the intensity perception of an emotion did not depend on the emotion-encoder’s gaze direction when she/he belonged to the ethnic in-group of the emotion-perceiver. However, when the emotion-encoder belonged to the ethnic out-group of the emotion-perceiver, the intensity perception of an emotion depended on her/his gaze direction. In addition, we found a tendency that emotions were perceived as more pronounced by participants from the ethnic out-group than from the ethnic in-group, when solely focussing on the emotion perception in situations where the agents directly addressed the participants.

### Effects of ethnicity

Each agent showed a negative (anger) and a positive (happiness) emotion. Overall, the animations should hence be assessed neutrally, with half of the animations being assessed negatively and half of the animations being assessed positively. Analysis of the main effect of *agent’s ethnicity* revealed that this was only the case for the animations of European agents but not for the animations of Asian agents, which were assessed slightly positively. To further investigate this effect, an analysis of the interaction of *participant’s ethnicity* and *agent’s ethnicity* was conducted. Results revealed that the slightly positive assessment of Asian agents is a consequence of Chinese participants rating animations of Asian agents as more positive than German participants. This indicates a positivity-bias for Chinese participants towards agents of their ethnic in-group, which might be explained by the so-called “intergroup bias” [41]. According to Hewstone and colleagues [41], people from different groups show a systematic tendency (intergroup bias) to evaluate one’s own in-group members more positively than out-group members. Culture is a key moderator of this intergroup bias: People from more collectivistic cultures, such as China, show a greater intergroup bias than people from more individualistic cultures, such as Germany [41], [42]. This is in concordance with the findings of the present study. On the one hand, Chinese participants who scored higher than German participants on the subscale measuring collectivism assessed Asian agents more positively compared to German participants. On the other hand, German participants who scored higher than Chinese participants on the subscale measuring individualism did not differ from Chinese participants in their assessment of European agents. Additionally, the correlation analysis of *participants’ SCS indices* and the assessment of *Asian agents* supports this explanation: Results revealed that the more positive the SCS index of a participant was (i.e. the more agreement she/he showed for collectivistic items), the more positive she/he assessed Asian agents.

### Effects of gaze direction

We expected to find a significant interaction effect between *gaze direction* and *emotion*. Based on findings of previous studies [9]–[11], we hypothesized that participants would rate the valence of emotions more extremely when displayed in combination with direct gaze as compared to averted gaze. Although we did find a corresponding significant interaction effect, our results cannot be interpreted as a general enhancement of the valence perception of emotions in the presence of direct gaze. In fact, the analysis of the four-way interaction revealed that the effect is modulated by an underlying interaction with *participant’s ethnicity* and *agent’s ethnicity*. Therefore, the hypothesis that emotions are generally perceived as more pronounced when expressed with direct gaze must be rejected. In fact, a more complex interplay between *gaze direction* and *ethnicity* needs to be taken into account.

### Interaction effects of gaze direction * ethnicity

Firstly, we focused on the effect of *ethnicity* on the appraisal of emotions displayed by agents expressing direct gaze or averted gaze. We expected that the effect of *gaze direction* on emotion perception (i.e. emotions shown with direct gaze are perceived as more pronounced than emotions shown with averted gaze) would be more pronounced when the participant and the agent belonged to the same ethnic group. Our results, however, suggest a different effect. We found a tendency that participants did not show any differences in the perception of emotions expressed with direct gaze as compared to averted gaze when the agent was an *ethnic in-group* member. This means that in most cases participants perceived anger and happiness just as pronounced when they were directly engaged with agents from their ethnic in-group as when they merely observed them engaging with someone else. This effect holds true for Germans assessing EUROPEANxHAPPINESS and EUROPEANxANGER, and for Chinese assessing ASIANxANGER.

On the basis of the observed effect in the other conditions, it might be expected that Chinese participants’ assessment of ASIANxHAPPINESS should be independent of gaze direction. Our results, however, indicate that Chinese assessed ASIANxHAPPINESSxDIRECT as more positive than ASIANxHAPPINESSxAVERTED. Interestingly, there is literature on differences in display rules between people from collectivistic and individualistic cultures [14], [38] which might explain the lack of such an effect in this condition. Ekman and Friesen [43] proposed that display rules help people manage and adjust emotional expressions depending on situational demands and social circumstances. It has been shown that there are differences in display rules between people from collectivistic and individualistic cultures [14], [38]. According to Matsumoto [44], people from collectivistic cultures show more positive emotional expressions towards ethnic in-group members in direct interactions than people from individualistic cultures. In addition, he demonstrated that display rules predict persons’ appropriateness ratings of the display of certain emotional expressions in social interactions. Based on these findings, one would expect Chinese participants to deem the expression of positive emotions towards ethnic in-group members particularly appropriate in direct interactions. Furthermore, this might result in Chinese preferring the display of positive emotions towards ethnic in-group members when expressed in combination with direct gaze as compared to averted gaze. This is exactly what we found in the present study: Chinese participants rated ASIANxHAPPINESSxDIRECT as more positive than ASIANxHAPPINESSxAVERTED. Results of the correlation analysis of *participants’ SCS indices* and the assessment of ASIANxHAPPINESSxDIRECT support this explanation: The more positive the SCS index of a participant was (i.e. the more agreement she/he showed for collectivistic items), the more positive she/he assessed ASIANxHAPPINESSxDIRECT. However, the presented explanation of this finding remains tentative. Thus, future research is required to investigate in detail whether distinct display rules applied by people from collectivistic cultures are indeed responsible for this difference.

Interestingly, when focussing on encounters with *ethnic out-group* members, we found a substantial influence of gaze direction: Participants assessed both emotional displays as more pronounced when agents from the ethnic out-group expressed them with direct gaze as compared to averted gaze. This implies that anger was assessed more negatively and happiness was assessed more positively when participants had the feeling of being directly engaged with an ethnic out-group member. On the other hand, when the ethnic out-group member expressed averted gaze, participants perceived both emotions as less pronounced.

In conclusion, except for Chinese assessing ASIANxHAPPINESS, we observed that the valence perception stayed the same when participants observed ethnic in-group members express emotions with direct gaze as compared to averted gaze. However, when participants observed ethnic out-group members express emotions with averted gaze as compared to direct gaze the intensity perception decreased. These results suggest that *gaze direction* has a differential influence on in-group and out-group dynamics during emotion perception in cross-cultural interactions.

Secondly, we focused on the effect that *agent’s ethnicity* has on the emotion appraisal of German and Chinese participants. We found no significant difference between Germans and Chinese in the emotion appraisal of agents expressing averted gaze. However, when participants were gazed at directly, in two out of four conditions participants from the ethnic out-group perceived the emotional expressions as more pronounced than participants from the ethnic in-group. Firstly, Germans assessed ASIANxANGERxDIRECT as more negative than Chinese. Secondly, Chinese assessed EUROPEANxHAPINESSxDIRECT as more positive than Germans. In the third condition (EUROPEANxANGERxDIRECT), although not significant, a tendency towards this effect was observable. Results indicated that Chinese showed a tendency to assess EUROPEANxANGERxDIRECT more negatively than Germans. Only in the fourth condition (ASIANxHAPPINESSxDIRECT), there was neither a significant effect nor a tendency towards such an effect. Based on the observed tendencies in the other three conditions, one would expect that Germans would assess ASIANxHAPPINESSxDIRECT more positively than Chinese. However, no such effect was found. Germans and Chinese did not differ in their assessment of ASIANxHAPPINESSxDIRECT. We presume that the positivity bias Chinese show towards agents of their own ethnicity [41] and their preference for the expression of positive emotions towards ethnic in-group members [14], [44] are responsible for the fact that Chinese assessed ASIANxHAPPINESSxDIRECT as positive as Germans.

## Conclusions

Our findings show that direct gaze as compared to averted gaze increases and emphasizes the perception of emotions shown by ethnic out-group members. However, except for Chinese assessing ASIANxHAPPINESS, gaze direction does not influence the intensity perception of emotions shown by ethnic in-group members. These results suggest that gaze direction has a differential influence on ethnic in-group and ethnic out-group dynamics during emotion perception in cross-cultural interactions. Furthermore, when focussing on direct gaze, we observed a tendency that emotions were perceived as more pronounced by ethnic out-group members than by ethnic in-group members. This could indicate that in direct encounters (indicated through direct gaze) ethnic out-group dynamics might be more important than ethnic in-group dynamics during emotion perception.

Overall, our findings were more complex than initially hypothesized. The results showed a strong interaction between *ethnicity* and *gaze direction*. To what degree, however, ethnicity influences the effect that gaze direction exerts over emotion perception seems to depend on underlying cultural constructs. We assumed that certain display rules and a greater intergroup bias for members of collectivistic cultures might have influenced the observed interaction effect. However, these speculations remain tentative, and future research should directly address the influence certain cultural constructs have on the interaction of ethnicity and gaze direction.
